# Digital Twin-Based Virtual Sensor Data Prediction and Visualization Techniques for Smart Swine Barns

**DOI:** 10.3390/s25247690

**Published:** 2025-12-18

**Authors:** Hyeon-O Choe, Meong-Hun Lee

**Affiliations:** 1 Low-Carbon Agriculture-Based Smart Distribution Research Center, Sunchon National University, Suncheon 57922, Republic of Korea; wishind@scnu.ac.kr; 2 Department of Convergence Biosystems Mechanical Engineering, Sunchon National University, Suncheon 57922, Republic of Korea

**Keywords:** smart swine barns, digital twin, virtual sensor, time-series prediction, hybrid model

## Abstract

To address the limitations of sensor deployment and high maintenance costs in smart swine barns, this study proposes a digital twin (DT)-based virtual sensor prediction and visualization method. Spatial constraints and harsh barn environments often cause sensor blackout zones, hindering precise environmental monitoring. To overcome these challenges, a virtual sensor was defined at the central position between Zone 1 and Zone 2, and its data were generated using a hybrid model that combines inverse distance weighting (IDW)-based spatial interpolation with long short-term memory (LSTM)-based time-series prediction. The proposed method was evaluated using 34,992 datasets collected from January to August 2025. Performance analysis demonstrated that the hybrid model achieved high prediction accuracy, particularly for variables with strong spatial heterogeneity, such as carbon dioxide (CO_2_) and ammonia (NH_3_), with overall coefficients of determination (R^2^) exceeding 0.95. Furthermore, a Web-based graphics library (WebGL) digital twin visualization environment was developed to intuitively observe spatiotemporal changes in sensor data. The system integrates sensor placement, risk-level assessment, and time-series graphs, thereby supporting users in real-time environmental monitoring and decision-making. This approach improves the precision and reliability of smart barn management and contributes to the stabilization of farm income.

## 1. Introduction

### 1.1. Challenges of Smart Swine Barns Environments and Need for Improved Monitoring

In recent years, Korea’s livestock industry has increasingly faced challenges related to aging workforce, labor shortages, animal welfare, and greenhouse gas emissions. To address these challenges, the transition to ICT-based smart swine barns has become essential [1]. Smart barns enable the simultaneous management of productivity and environmental conditions by monitoring and controlling temperature, humidity, and atmospheric gases in real time, and are regarded as a key driver of the digital transformation in livestock farming [2]. However, in practice, spatial constraints inside barns limit sensor installation, resulting in blackout zones that hinder comprehensive data collection [3].

Furthermore, in outdated barns, physical damage to sensors frequently occurs due to dust and humidity. Dust accumulation on sensor surfaces can cause malfunctions and shorten their lifespan, whereas high concentrations of harmful gases and particulates make it difficult to maintain the reliability and durability of physical sensors, thereby increasing maintenance costs [4]. For instance, ammonia sensors cost between KRW 400,000 and 1,500,000 [5], yet their operational lifespan is short under harsh conditions, resulting in frequent replacements and higher maintenance expenses. Additional infrastructure—such as electrical wiring, data acquisition devices, and control systems—is also required to install sensors and collect data, further increasing financial burden.

### 1.2. Concept and Necessity of Digital Twin-Based Virtual Sensors

As an alternative to these limitations, virtual sensors have attracted increasing attention. Based on real-world data, virtual sensors estimate values at unmeasured locations using modeling or interpolation techniques, thereby addressing blackout zones and improving the completeness of environmental monitoring [6]. They can enhance data accuracy while reducing installation and operation costs, contributing to more efficient management of smart barns.

In particular, the recently highlighted digital twin technology creates a virtual replica of a physical system and enables real-time linkage for monitoring, simulation, prediction, and optimization [7]. Its adoption in agriculture and livestock industries has been rapidly expanding, with growing applications in barn environment monitoring, animal behavior analysis, disease prevention, and feed management optimization [8,9]. Digital twins combine physical and virtual sensors to estimate barn-wide conditions more accurately, offering potential benefits for productivity improvement, cost reduction, and animal welfare.

However, research integrating virtual sensors and digital twins for predictive and visual applications in smart swine barns environments remains limited. Previous studies have mainly focused on optimizing sensor networks or designing digital twin architectures for smart barns [10,11], whereas studies combining real sensor data-based virtual sensing with digital twin visualization are still in their early stages. Therefore, this study proposes a methodology for digital twin-based sensor data prediction and visualization by integrating physical and virtual sensors in a smart barn environment. Specifically, virtual sensors (temperature, humidity, CO_2_, and ammonia) were defined at the central position between Zone 1 and Zone 2 using real sensor data, and their values were estimated through a combination of time-series prediction and spatial interpolation techniques. A digital twin-based visualization platform incorporating the virtual sensor data was then developed to complement blackout zones and enhance the precision of environmental monitoring.

The remainder of this paper is structured as follows. Section 2 reviews the research trends and related literature on virtual sensors and digital twin technology. Section 3 describes the methodology for defining virtual sensors based on smart barn data and generating their data using time-series prediction and spatial interpolation techniques. Section 4 presents the experimental results by applying the proposed method to real barn data, verifying virtual sensor performance, and constructing a WebGL-based digital twin visualization environment. Finally, Section 5 concludes the study and discusses future research directions.

### 1.3. Related Research

Building on the challenges and technological needs discussed in the previous sections, this subsection examines recent research trends related to digital twin applications in smart agriculture, as well as virtual sensor techniques and environmental data-prediction methodologies used in agricultural and livestock settings. Through this review, we position the proposed approach within existing literature, clarify its distinctions from prior work, and highlight the necessity of integrating virtual sensor technology with digital twin-based predictive and visualization frameworks for smart barn environments.

#### 1.3.1. Digital Twin Cases in Smart Agriculture

In recent years, digital twin technology has emerged as a key innovation not only in the industrial and manufacturing sectors but also in smart agriculture and livestock management. A digital twin enables real-time reflection of the physical environment and predictive simulation through bidirectional data flow between physical systems and their virtual counterparts. This capability significantly enhances agricultural productivity, data-driven decision-making, and environmental adaptability [12,13,14,15].

Digital twins in smart agriculture have evolved in three main directions. First, greenhouse and crop growth digital twins optimize environmental control—temperature, humidity, CO_2_, and light intensity—and perform crop growth simulations. Second, livestock environment digital twins replicate animal living conditions, behaviors, and health data in virtual space for real-time monitoring. Third, agricultural equipment and infrastructure digital twins aim to improve energy efficiency and reduce maintenance and operational costs [16,17,18,19].

**Figure 1:**
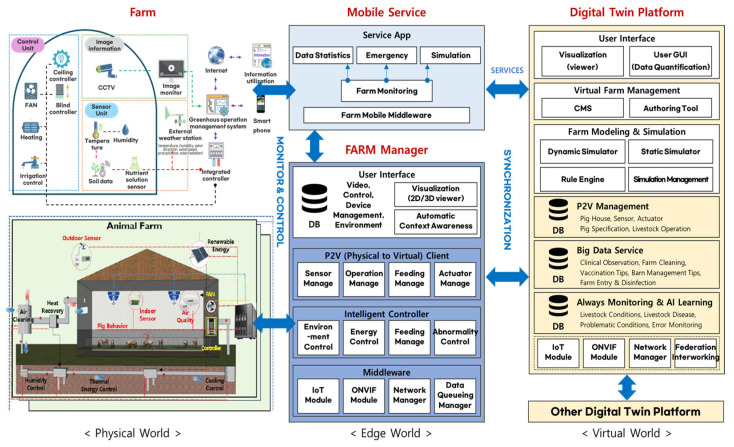
Hierarchical digital twin architecture for agriculture and livestock.

Figure 1, adapted from Kim et al. (2022) [20], illustrates an integrated hierarchical digital twin architecture designed for agriculture and livestock.

This architecture consists of three layers: the Physical World, Edge World, and Virtual World. In the Physical World, sensors and devices, such as temperature, humidity, CO_2_, and ammonia sensors, as well as controllers and ventilation equipment, collect environmental data from within the barn. In the Edge World, the data are preprocessed and stored locally through a gateway, then transmitted to the cloud-based Virtual World. The Virtual World utilizes the collected data to conduct 3D simulations, develop AI-based predictive models, and test environmental control scenarios, which are then used to update and manage the physical environment in real time.

The core of this layered structure is the real-time feedback loop between the physical system and virtual model. Sensor data are transmitted to the digital twin platform for visualization and analysis, and the resulting insights are fed back to the physical system for adaptive control. This bidirectional loop enables the implementation of intelligent control systems that can respond immediately to environmental changes within barns—such as rapid temperature increases or ventilation failures [21,22,23,24,25].

The proposed digital twin-based virtual sensor system extends this hierarchical architecture to a smart barn environment. Based on real sensor data from Zones 1 and 2 in the Physical World, the system predicts and visualizes the virtual sensor node data within the Virtual World, thereby overcoming the spatial limitations of sensor installation. Through this approach, an intelligent livestock management framework was established, enabling real-time data-driven environmental simulation and spatial prediction.

#### 1.3.2. Simple Interpolation-Based Method

The most fundamental approach to implementing virtual sensors involves the use of simple interpolation techniques. Representative methods include linear interpolation, inverse distance weighting (IDW), and Kriging, all of which estimate unobserved values based on the distance between sensors. These methods are computationally simple and intuitive [26]. For example, the value at the midpoint between Zone 1 and Zone 2 can be estimated as either the simple average of both sensors or a distance-weighted sum. However, such methods cannot sufficiently capture temporal patterns or nonlinear environmental variations and are vulnerable to abrupt changes caused by external factors [27,28].

**Figure 2:**
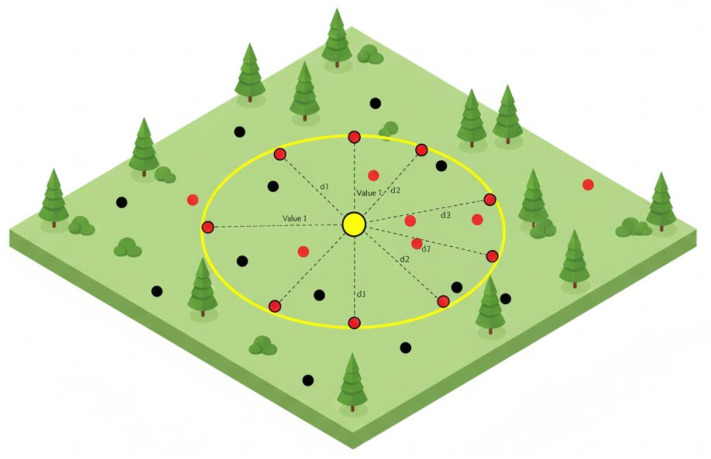
Principle of the inverse distance weighting (IDW) interpolation method. Adapted from Cloud’s Daily Report (2023) [30].

As shown in Figure 2, IDW offers the advantage of simplicity and intuitive computation, making it suitable for quickly identifying spatial trends in a limited sensor network environment. However, in agricultural and livestock environments, physical factors, such as airflow, heat distribution, and humidity gradients exhibit nonlinear spatial behavior, making it difficult for IDW alone to accurately reflect real-world variability. Therefore, while simple interpolation functions as a useful preliminary step for basic spatial estimation, it has inherent limitations for predicting dynamic environmental conditions [29].

For instance, temperature or ammonia concentration inside a barn can change rapidly over time depending on fan operation, ventilation pathways, and livestock movement. Such non-stationary spatial patterns are difficult to model using fixed distance-based IDW. Consequently, recent studies have extended these methods into spatio-temporal interpolation by integrating time-series analysis or deep learning models to better represent the complex dynamics of environmental changes.

#### 1.3.3. Time Series-Based Forecasting Model

Time-series forecasting models, which consider temporal continuity, have emerged as an effective means of overcoming the limitations of simple interpolation techniques. Traditionally, statistical approaches, such as Autoregressive Integrated Moving Average (ARIMA) and Vector Auto Regression (VAR) have been used; however, in recent years, deep learning-based models, such as Long Short-Term Memory (LSTM), Gated Recurrent Unit (GRU), and Temporal Convolutional Network (TCN) have been widely applied for sensor data prediction [31]. These models can learn long-term dependencies and capturing nonlinear changes in data, while maintaining robustness against sensor malfunctions and noise. However, one notable drawback is that spatial location information is not directly incorporated into their structure.

#### 1.3.4. Space-Time Fusion Model

Simple spatial interpolation and time-series prediction techniques capture only one aspect of variation—spatial or temporal—and thus are limited when applied to systems, such as smart swine barns, where spatial heterogeneity and temporal nonlinearity coexist. To address this limitation, recent studies have actively proposed spatio-temporal fusion approaches that jointly model both dimensions.

A spatio-temporal fusion model consists of three main components: spatial–temporal feature extraction, an encoder, and a decoder. Input data are first transformed into a spatial feature map using a convolutional neural network (CNN) to represent spatial characteristics and temporal patterns simultaneously. The sequence is then processed through a Transformer, LSTM, or Temporal Graph Attention network to learn temporal dependencies [32,33,34,35]. In the decoder stage, the model integrates spatial and temporal patterns to reconstruct missing spatial data or predict future values.

**Figure 3:**
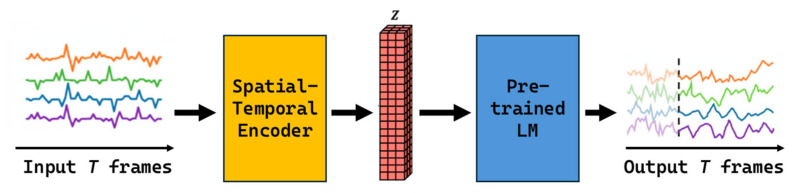
Architecture of the spatio-temporal Transformer model based on space–time fusion. Adapted from Mohamed et al. (2021) [37].

As shown in Figure 3, the Spatio-Temporal Transformer is a representative structure for space–time fusion modeling. It converts input time-series data into spatio-temporal embeddings and learns spatial–temporal correlations through a multi-head self-attention mechanism that incorporates positional information [36].

In environments, such as swine barns, where temperature, humidity, CO_2_, and NH_3_ interact dynamically, this structure captures inter-variable influences while maintaining spatio-temporal continuity. The key feature of a spatio-temporal fusion model is its ability to handle interactions between temporal and spatial axes within a unified framework. By learning interdependencies among spatially adjacent sensor nodes while simultaneously reflecting temporal variations—such as diurnal temperature shifts or seasonal humidity fluctuations—the model achieves significantly higher generalization performance compared to single-axis models.

#### 1.3.5. Hybrid Approach

A hybrid approach that combines spatial interpolation with time-series modeling has also been explored in recent studies. In this approach, spatially interpolated values—derived from methods, such as IDW or Kriging—are first computed, and then temporal patterns are refined using models, such as LSTM or Transformer [38]. This method enhances prediction accuracy by simultaneously incorporating spatial and temporal characteristics, although it introduces greater model complexity and requires additional preprocessing steps.

Among the various virtual sensor generation methods discussed above, this study adopts a hybrid approach that integrates time-series forecasting with spatial interpolation. The aim is to compensate for sensor blackout zones in smart barn environments and ensure stable data estimation even when physical sensors fail or data are missing. While simple interpolation techniques are computationally efficient, they cannot adequately represent dynamic environmental variations; conversely, purely deep learning-based time-series models fail to explicitly account for spatial constraints. The proposed approach maximizes the strengths of both methods by accurately capturing temporal dynamics while simultaneously considering spatial relationships. In addition, contrary to previous studies that have primarily focused on verifying model performance, this study presents an integrated framework that applies the hybrid model within a WebGL-based digital twin visualization platform for practical real-time environmental monitoring.

## 2. Materials and Methods

This section describes the overall research procedure for predicting and visualizing virtual sensor data in a digital twin-based smart swine barns environment. The study aims to implement a digital twin framework that integrates the Physical World and the Virtual World using real barn data, while designing and validating a hybrid prediction model that reflects spatio-temporal correlations.

### 2.1. Data Collection and Preprocessing

The experimental barn was a single-story finisher pig house located in Jeollanam-do, Korea. The internal floor area measured approximately 9 m in width and 25 m in length. A total of 30 fattening pigs (*Sus scrofa domesticus*) were housed in the facility under a breeding-to-farrowing management system. The interior was operated as two environmental zones corresponding to Zones 1 and 2, and the animals were group-housed within these zones throughout the monitoring period.

Environmental data collected from Zones 1 and 2 of a smart swine barns located in Jeollanam-do, Korea, were used for analysis. As summarized in Table 1, each zone was equipped with sensors measuring temperature (°C), humidity (%), carbon dioxide (CO_2_, ppm), and ammonia (NH_3_, ppm). Measurements were taken at approximately 10-min intervals. The data collection period spanned from 1 January–31 August 2025, resulting in a total of approximately 34,900 recorded entries.

Some missing and anomalous values were found in the collected metadata. Missing values, which accounted for approximately 1.5% of the dataset, were primarily caused by sensor communication errors or temporary power instability. Linear interpolation was applied to preserve the continuity of the time-series data and compensate for missing entries. Extreme values (e.g., temperatures below −50 °C or humidity levels of 0% or 120%) were identified as sensor malfunctions and removed from the dataset.

The final refined dataset contained 34,992 data points after preprocessing. These were used for defining virtual sensors and training the prediction model. The processed dataset also functioned as input for virtual sensor generation, performance verification, and construction of the digital twin visualization environment.

The environmental variables were monitored using commercially available sensors widely adopted in livestock production facilities. Temperature and relative humidity were measured using the SHT3x-DIS digital sensor (Sensirion AG, Stäfa, Switzerland), which provides factory-calibrated and temperature-compensated outputs with high accuracy. CO_2_ concentrations were recorded using the MH-Z14A NDIR sensor (Winsen Electronics, Zhengzhou, China), offering a measurement range up to 5000 ppm with integrated temperature compensation to ensure stability under barn conditions. NH_3_ concentrations were monitored using the ME3NH3 (Winsen Electronics, Zhengzhou, China) electrochemical sensor, which is suitable for detecting low-level ammonia emissions typically observed in pig housing environments. The specifications of these sensors, including measurement ranges and accuracies, are summarized in Table 2. Prior to installation, all sensors were calibrated according to manufacturer guidelines, and additional stability checks were conducted during the monitoring period to maintain reliable performance in the field.

The barn was divided into two environmental zones (Zone 1 and Zone 2) along the longitudinal direction of the facility. This zoning followed the existing management layout of the finisher pens rather than an arbitrary spatial split.

In the experimental finisher pig house, the animals were group-housed in fixed pens, and their movement was confined within each pen. As a result, animal behavior mainly influenced the internal environment through gradual changes in metabolic heat and gas emission rather than through abrupt relocation between zones. The environmental sensors were installed near the ceiling along the central axis of the barn, so that they measured well-mixed air conditions representing each zone rather than local point fluctuations near the animals. Therefore, short-term movements of individual pigs were not found to cause distinct spikes in the recorded temperature, humidity, CO_2_, or NH_3_ data at the 10 min sampling interval; instead, animal activity contributed to slow temporal variations that were naturally captured by the hybrid prediction model.

### 2.2. Defining and Modeling Virtual Sensors

Because the two zones exhibit gradual rather than abrupt environmental transitions, the midpoint between Zone 1 and Zone 2 represents the intermediate region where air mixing occurs along the central ventilation path. Placing the virtual sensor at this location allowed the model to capture the spatial gradient between the two zones while avoiding local noise generated near pen-level activity. This placement provides a physically meaningful target point for interpolating spatial information and enhances the reliability of spatio-temporal prediction.

**Figure 4:**
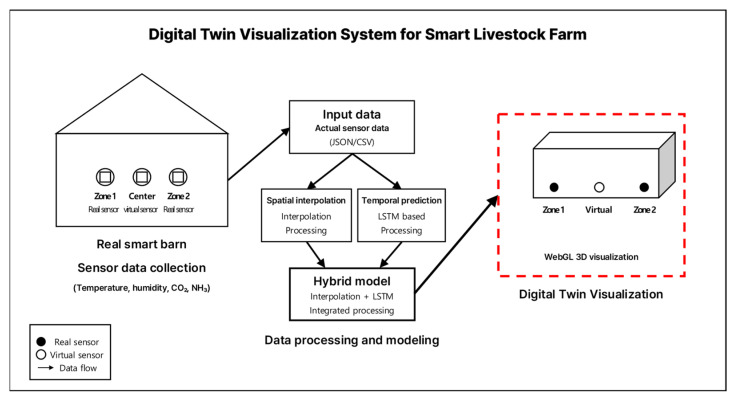
Conceptual diagram of virtual sensor placement.

**Figure 5:**
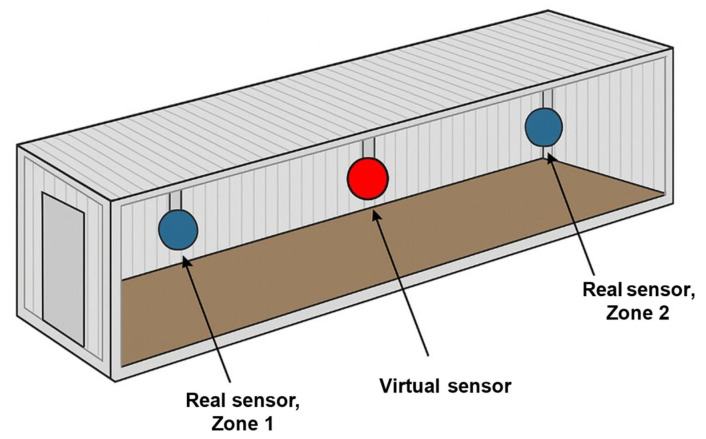
Physical and virtual sensor layout.

Figure 4 and Figure 5 illustrate the virtual sensor placement and conceptual diagram used in this study. Sensors were installed in Zones 1 and 2 within the barn, and a virtual sensor was defined at the midpoint between the two zones. Because it is often impractical to densely install sensors in real barn environments, the virtual sensor was introduced to compensate for blackout zones and enhance the overall precision of spatial environmental monitoring.

The virtual sensor was designed to measure the same four variables as the physical sensors: temperature (°C), humidity (%), CO_2_ (ppm), and NH_3_ (ppm). The virtual sensor values were generated using a hybrid approach that combines spatial interpolation with time-series prediction.

First, the value at the central position between Zones 1 and 2 was estimated using the measured data from both zones, as expressed in Equation (1). Spatial interpolation was performed using either linear interpolation or IDW. The virtual sensor value $V_{vs}(t)$ at time t was calculated as the distance-weighted average of the measured values from the two adjacent zones, as follows:

(1) $$V_{vs}\left(t\right)=\left(d^{2}/(d^{1}+d^{2})\right)*V^{1}\left(t\right)+\left(d^{1}/(d^{1}+d^{2})\right)*V^{2}\left(t\right)$$

Here, $V_{1}(t)$ and $V_{2}(t)$ denote the sensor values in Zones 1 and 2, respectively, and $d_{1}$ and $d_{2}$ represent the distances from the virtual sensor to each zone.

As the virtual sensor was placed at the midpoint, $d_{1}=d_{2}$, and Equation (1) can be simplified into a simple average as in Equation (2):

(2) $$V_{vs}\left(t\right)=\left(V^{1}\left(t\right)+V^{2}\left(t\right)\right)/2$$

The internal operations of the LSTM network used in this study are defined in Equations (3a)–(3f). At each time step t, the LSTM computes four gate values and updates its cell and hidden states. The input gate $i_{t}$ regulates how much new information enters the memory cell (3a), while the forget gate $f_{t}$ determines how much of the previous cell state $c_{t-1}$ is retained (3b). A candidate cell state ${\tilde{c}}_{t}$ is generated using the hyperbolic tangent activation function (3c), and the final cell state $c_{t}$ is updated by combining the retained memory and new candidate information (3d). The output gate $o_{t}$ controls how much of the cell state contributes to the hidden state (3e), and the final hidden state $h_{t}$ is obtained by modulating the activated cell state (3f).

(3a) $$i_{t}=\sigma \left(W_{i}x_{t}+U_{i}h_{t-1}+b_{i}\right)$$

(3b) $$f_{t}=\sigma \left(W_{f}x_{t}+U_{f}h_{t-1}+b_{f}\right)$$

(3c) $${\tilde{c}}_{t}=tanh\left(W_{c}x_{t}+U_{c}h_{t-1}+b_{c}\right)$$

(3d) $$c_{t}=f_{t}⊙c_{t-1}+i_{t}⊙{\tilde{c}}_{t}$$

(3e) $$o_{t}=\sigma \left(W_{o}x_{t}+U_{o}h_{t-1}+b_{o}\right)$$

(3f) $$h_{t}=o_{t}⊙tanh\left(c_{t}\right)$$

Here, $x_{t}$ denotes the input at time t, $h_{t-1}$ is the previous hidden state, and $c_{t}$ is the memory cell state. $W_{*}$ and $U_{*}$ represent learnable weight matrices, $b_{*}$ are the bias terms, σ(⋅) is the sigmoid activation function, tanh(⋅) is the hyperbolic tangent function, and ⊙ denotes element-wise multiplication. This formulation enables the LSTM to capture long-term dependencies and nonlinear temporal patterns essential for environmental prediction tasks.

While simple interpolation techniques are computationally efficient and intuitive, they fail to capture the temporal dynamics of environmental variations within barns. Conversely, time-series models, such as LSTM are powerful in learning nonlinear patterns and long-term dependencies but do not directly incorporate spatial relationships, thereby overlooking the spatial structure of sensor placement.

To address these limitations, this study employed a hybrid approach that integrates spatial interpolation with time-series prediction.

The interpolated value $V_{vs}(t)$ from the spatial step functioned as the input to the LSTM model, which then produced the final predicted value ${\tilde{V}}_{vs}(t+1)$ as expressed in Equation (4):

(4) $${\tilde{V}}_{v}s\left(t+1\right)=f_{\theta }\left(V_{v}s\left(t-k+1\right),\ldots ,V_{v}s\left(t\right)\right)$$

Here, $f_{\theta }$ denotes the learning parameter of the LSTM model, and k represents the input sequence length. The hybrid model first estimated the virtual sensor’s initial value through spatial interpolation between Zones 1 and 2, and then applied the LSTM model to learn temporal patterns and correct residual errors. This process allows the model to simultaneously consider spatial correlations and temporal continuity, thereby improving prediction accuracy and stability compared with single-method approaches.

Notably, contrary to prior studies that focused solely on model performance comparison, this study verified the results through integration with a WebGL-based digital twin visualization platform. This approach enabled intuitive observation of how the virtual sensor values were represented within the actual environment, providing a practical foundation for smart barn operators to assess data reliability in real time and support data-driven control decisions—representing a key distinction from existing studies.

### 2.3. Digital Twin Visualization Environment

**Figure 6:**
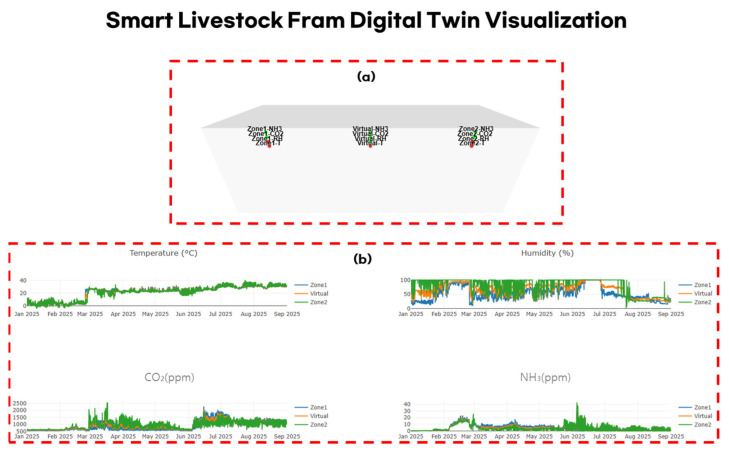
Smart swine barns digital twin visualization environment and sensor dashboard. (**a**) WebGL-based 3D barn model with spatially mapped sensor nodes and risk-level color coding; (**b**) Interactive sensor dashboard showing environmental variables and time-series graphs.

In this study, a WebGL-based web environment was developed to visualize the indoor conditions of the smart swine barns as a digital twin. The visualization system was constructed using the Three.js (version r152) and Plotly.js (version 2.27.0) libraries and comprise four main components: three-dimensional spatial modeling, dynamic updates of sensor data, visual changes according to risk-level assessment, and time-series graph representation. Figure 6 illustrates the virtual environment and sensor dashboard of the smart farm digital twin.

To simplify the spatial configuration of the barn, a rectangular container structure with a length of 25 m, width of 9 m, and height of 5 m was modeled. The top (ceiling) and front faces were rendered transparent to allow visual observation of the internal sensor nodes, while the remaining surfaces were shaded in gray tones to provide a realistic spatial impression of the barn.

Sensor nodes were positioned at three locations—Zone 1, the virtual sensor, and Zone 2—and each position was designed to include four sensors measuring temperature, humidity, carbon dioxide, and ammonia. The nodes were represented as spheres, and each was labeled using CSS2DRenderer with tags, such as “Zone1-T” and “Virtual-CO_2_” such that the sensor type and location could be intuitively identified.

Data were loaded from a pre-collected JSON file (swine_barns_data_full.json) and updated frame by frame in chronological order. Sensor values were dynamically color-coded in real time according to the environmental criteria defined in this study.

The criteria were set as follows: temperature was defined as comfortable at 18–21 °C under normal conditions or 28–30 °C during summer; humidity between 50% and 70%; CO_2_ concentration below 3000 ppm; and ammonia concentration below 20 ppm were considered normal. Sensor nodes were displayed in green under normal conditions, yellow for warning states, and red for danger states, enabling intuitive identification of the risk level in each zone.

Using Plotly.js, independent time-series graphs were generated for temperature, humidity, carbon dioxide, and ammonia. Each graph simultaneously displays the measured values from Zone 1, the virtual sensor, and Zone 2, allowing comparative analysis between predicted results and actual sensor readings. This allows observation of both spatial visualization and temporal variation patterns, enabling quantitative assessment of how environmental changes affect livestock growth.

The proposed digital twin visualization environment integrates spatial configuration and temporal changes to comprehensively represent environmental conditions and risk levels within the barn. By combining risk-level visualization of sensor data with graph-based time-series analysis, the system allows managers to intuitively detect abnormal conditions in specific zones and at specific points in time.

Beyond providing a graphical representation of the swine barns layout, the WebGL-based digital twin environment offers practical benefits for livestock management. Because the visualization operates in a standard web browser without requiring dedicated software, producers and facility managers can access real-time environmental conditions from any device. The ability to render spatially continuous distributions of temperature, humidity, CO_2_, and NH_3_ enables users to intuitively identify risk areas that may not be apparent from raw sensor values alone. In addition, the platform can be extended to integrate control logic or alert functions, allowing the visualization to serve as a front-end interface for real-time decision-making and operational adjustments. These practical features enhance the usability and applicability of the proposed system in routine swine barns management.

### 2.4. Experimental Design and Evaluation Methods

This study designed a systematic experiment to verify the performance of the virtual sensor-based data generation method and evaluate the applicability of the digital twin visualization environment. The experiments consisted of three scenarios.

First, the normal condition scenario aimed to verify the prediction accuracy of the model under stable environmental conditions by predicting the virtual sensor values at the central zone based on the physical sensor data from Zones 1 and 2.

Second, the sensor loss scenario simulated real-world sensor failure by intentionally removing data from either Zone 1 or Zone 2. This scenario was designed to evaluate the capability of the virtual sensor to compensate for missing data when sensor malfunctions occur within the barn.

Third, the abrupt environmental change scenario assumed situations where temperature, humidity, CO_2_, and NH_3_ concentrations change rapidly within the barn, to verify the model’s ability to track nonlinear and sudden environmental variations.

The dataset used for the experiments consisted of 34,992 records collected from Zones 1 and 2 between 1 January and 31 August 2025. Each record contained four environmental variables: temperature (°C), humidity (%), CO_2_ concentration (ppm), and NH_3_ concentration (ppm).

Considering the temporal characteristics of the data, it was divided into training data (January–June 2025, 26,244 records, 75%) and validation data (July–August 2025, 8748 records, 25%). This time-based split reflects a practical scenario in which accumulated past data are used to predict future conditions in real barn operations.

For comparison, three baseline methods were implemented: simple mean interpolation, linear regression, and pure LSTM modeling. Simple mean interpolation represents the most basic spatial estimation method, while linear regression captures linear relationships among variables using a statistical approach. Pure LSTM focuses on learning temporal patterns and models time-based changes but does not incorporate spatial relationships. In contrast, the proposed hybrid interpolation–LSTM model combines spatial interpolation with temporal pattern learning, achieving both accuracy and robustness.

To quantitatively evaluate performance, the mean absolute error (MAE), root mean square error (RMSE), and coefficient of determination (R^2^) were used as evaluation metrics. MAE represents the average absolute difference between predicted and actual values, RMSE measures the square root of the mean of squared errors and is sensitive to large deviations, and R^2^ indicates how well the model explains the variance of the observed data.

## 3. Results

### 3.1. AI Model Evaluation Results

To verify the prediction performance of the virtual sensor, three models were applied: the spatial interpolation-based IDW model, time-series LSTM model, and hybrid model combining IDW and LSTM. Model performance was evaluated using RMSE, MAE, and R^2^ metrics.

Table 3 presents the prediction accuracy for each variable. For temperature (T_virtual), both the LSTM and hybrid models showed a high level of agreement with the actual observed values (R^2^ = 0.970 and 0.959, respectively), with RMSE values of 0.438 and 0.515. For humidity (RH_virtual), the LSTM achieved the best results (RMSE = 1.805, R^2^ = 0.978), while the hybrid model demonstrated comparable performance (RMSE = 1.887, R^2^ = 0.976). For CO_2_ prediction, the hybrid model achieved slightly higher accuracy (RMSE = 22.733, R^2^ = 0.981) than the LSTM (RMSE = 23.348, R^2^ = 0.980). For NH_3_, the hybrid model also outperformed the LSTM, with RMSE = 0.340 and R^2^ = 0.964, compared with RMSE = 0.392 and R^2^ = 0.952 for the LSTM.

**Figure 7:**
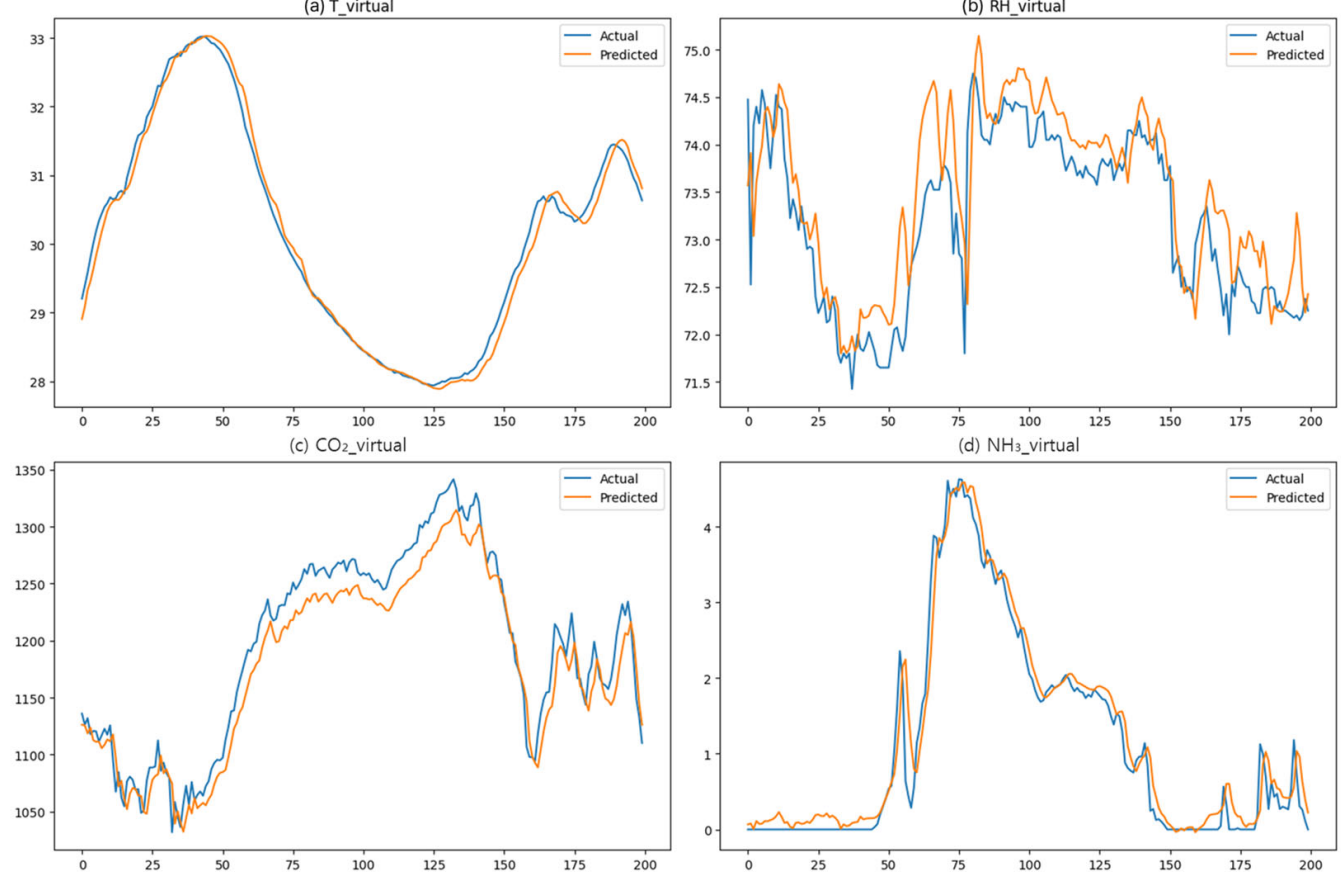
Comparison between actual and predicted values at the virtual sensor location. (**a**) Temperature; (**b**) Relative humidity; (**c**) CO_2_ concentration; (**d**) NH_3_ concentration.

Figure 7 presents a visual comparison between actual and predicted values. Across all variables, the predicted values closely followed the actual patterns, and the hybrid model in particular demonstrated stable tracking during periods of rapid fluctuations in CO_2_ and NH_3_ concentrations. This result experimentally confirms that the hybrid approach incorporates both spatial contexts and temporal patterns.

**Figure 8:**
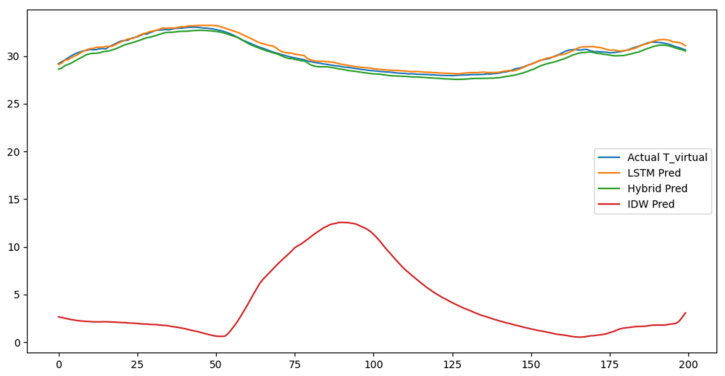
Comparing prediction curves (Actual vs. LSTM vs. Hybrid vs. IDW).

Figure 8 compares the actual temperature values at the virtual sensor location—calculated from the measured data of Zones 1 and 2—with the results of the three prediction models (IDW, LSTM, and Hybrid).

The IDW method, which estimates values through simple distance-based interpolation, failed to capture the overall temporal trend and showed excessive deviations in intermediate sections. This occurred because IDW does not account for temporal dynamics, resulting in poor representation of short-term temperature rises or drops.

In contrast, the LSTM model reproduced the general shape of the actual temperature variation curve by learning temporal continuity and nonlinear fluctuation patterns. However, prediction lag occurred in certain sections, and the model could not account for the spatial influence of sensor locations.

The proposed hybrid model (IDW + LSTM) combined the strengths of both methods using the spatially interpolated initial values as inputs for LSTM-based temporal correction. The differences between the actual and predicted values were minimal, and the prediction curve consistently reproduced the actual temperature variation trends across the entire period.

Across the different scenarios in Figure 8, the performance differences among the three models can be interpreted based on their underlying computational characteristics. IDW relies solely on geometric distance; therefore, it responds immediately to changes in neighboring sensor values but cannot reflect temporal continuity, making it more sensitive to short-term fluctuations or inconsistencies caused by sensor drift. In contrast, the LSTM model smooths abrupt variations by learning temporal dependencies, which improves stability but can delay the model’s response to sudden environmental changes. The hybrid model integrates both spatial and temporal components, enabling it to adjust to abrupt changes while still maintaining continuity over time. This combined behavior explains why the hybrid framework shows the most balanced performance under both gradual and rapid environmental variations.

In quantitative evaluation, the hybrid model achieved an RMSE of 0.515 and R^2^ of 0.959, representing approximately a 3.4% improvement in accuracy over the standalone LSTM model. This result indicates that a model incorporating both spatial and temporal factors can estimate environmental changes inside the barn with greater precision.

### 3.2. Scenario-Specific Result Interpretation

Table 4 summarizes the performance comparison among the algorithms. The IDW method produced results quickly through simple computation, but its inability to reflect temporal patterns limited its applicability in real-world environments. In contrast, the LSTM model demonstrated strong performance in learning time-series patterns, while the hybrid model, which simultaneously considered spatial distribution and temporal dynamics, produced the most stable results for certain variables.

First, IDW interpolation offered the advantage of being simple and intuitive, and it provided reasonable estimates when the spatial gradient between Zones 1 and 2 was relatively smooth. However, because IDW relies solely on geometric distance, it cannot represent temporal dependencies or delayed responses to environmental changes. As a result, its performance degraded under more complex and dynamically changing barn conditions, where both spatial and temporal variability jointly influenced the environment.

Second, the LSTM-based time-series model captured nonlinear temporal patterns and short-term fluctuations using historical sequences from each sensor location. This approach was effective in modeling gradual changes and recurrent patterns over time. Nevertheless, because the LSTM operated independently on each sensor without explicit spatial structure, it tended to produce spatially averaged behavior and could not fully reflect environmental differences between zones or at unseen intermediate locations.

Third, the hybrid model (IDW + LSTM) integrated the strengths of both approaches by using IDW-based spatial estimates as inputs to the LSTM. This allowed the model to preserve spatial relationships between zones while simultaneously learning temporal dynamics, resulting in the most stable and accurate predictions at the virtual sensor location. The benefit of the hybrid framework was particularly evident under scenarios with both spatial heterogeneity and temporal variability, whereas in more spatially homogeneous conditions its advantage over the standalone LSTM model was smaller but still comparable in accuracy.

## 4. Discussion

Previous studies have emphasized that environmental monitoring in swine barns buildings is often hindered by spatial heterogeneity, limited sensor placement, and sensor degradation over time [39]. These issues lead to incomplete environmental representations and reduce the reliability of data-driven management systems. The present study ad-dresses these technical challenges by integrating spatial interpolation with temporal pre-diction to construct a virtual sensing framework capable of compensating for blackout zones and mitigating the effects of sensor drift. Similar virtual sensor and digital twin approaches have been reported in greenhouse monitoring and environmental control applications [40,41], but research specific to swine barns remains limited. Our findings extend these prior works by demonstrating that a hybrid spatial–temporal model can more accurately reconstruct unmeasured environmental variables in a swine barns setting characterized by rapid airflow variations and temperature gradients.

The experimental results demonstrate that the proposed hybrid interpolation–LSTM model provides more accurate estimations for variables characterized by strong spatial heterogeneity, particularly CO_2_ and NH_3_. These gases typically exhibit localized concentration gradients within swine barns environments due to animal activity, manure emission points, ventilation airflow, and structural constraints. Purely spatial methods such as IDW cannot fully represent these complex dispersion patterns, while LSTM alone lacks spatial context and therefore produces temporally consistent but spatially generalized predictions. By combining both approaches, the hybrid model leverages spatial priors from IDW and subsequently refines them through temporal learning, enabling it to better represent abrupt fluctuations and asymmetric spatial profiles. This integrated mechanism explains the superior performance observed for spatially sensitive variables.

The structural differences between the standalone LSTM model and the hybrid approach further highlight the importance of incorporating spatial information into the prediction process. While LSTM effectively captures nonlinear temporal dependencies across long sequences, it relies solely on historical patterns and cannot account for spatial variability between different zones. As a result, its predictions tend to converge toward averaged temporal behaviors, particularly when environmental conditions vary sharply over space. In contrast, the hybrid model conditions the temporal prediction on an initial spatial estimate, allowing the LSTM to learn deviations from spatial baselines rather than reconstructing the full spatio-temporal pattern from scratch. This leads to improved stability and responsiveness, especially under dynamic CO_2_ and NH_3_ conditions.

Several considerations must be addressed when applying the proposed model to real swine barns environments. The performance of spatial interpolation is influenced by the physical layout of the barn, the geometry of ventilation paths, and the distribution of animals; thus, model recalibration may be required when facility configurations change. In addition, the reliability of LSTM-based predictions depends on the availability of sufficient high-quality historical data. Noise introduced by sensor drift, communication delays, or intermittent failures could reduce predictive accuracy unless properly preprocessed. Furthermore, real-time implementation demands low-latency data processing and stable network infrastructure to ensure seamless integration with the digital twin interface. Addressing these practical constraints will be essential for scaling the proposed approach to commercial barn operations.

Compared with previous studies, the proposed method offers a more integrated framework that combines spatial interpolation, time-series modeling, and digital twin visualization. Earlier works have typically focused on either predictive modeling or digital twin design, but few have demonstrated a fully operational system capable of producing virtual sensor outputs while simultaneously supporting real-time monitoring. By validating the hybrid prediction model within an interactive 3D environment, this study bridges the gap between theoretical modeling and real-world applicability, providing a practical foundation for future digital twin-based swine barns management systems.

From an operational perspective, the use of virtual sensors can provide meaningful economic benefits in swine barns. In commercial pig houses, NH_3_ sensors—typically electrochemical devices with a service life of 8–12 months—are among the most frequently replaced components due to exposure to high humidity, dust, and corrosive gases. The ME3NH3 sensors used in this study generally cost between USD 60 and 120 per unit, and typical barn configurations require multiple sensors to monitor different zones. Replacing these sensors annually can therefore represent a recurring expense. By contrast, virtual sensors can estimate environmental conditions at additional locations without the need for new hardware. For a facility equipped with two physical gas sensors, the ability to substitute one or more of these with virtual sensors can reduce annual hardware replacement and maintenance costs by approximately 30–40%, depending on the facility layout and the number of installed devices. These results highlight the practical economic value of integrating virtual sensing into continuous monitoring systems for pig housing environments.

The WebGL-based visualization further strengthens the practical utility of the framework by enabling intuitive identification of environmental anomalies and facilitating remote monitoring through a browser-based interface. Such features allow the digital twin to function not only as a prediction tool but also as a decision-support component within daily swine barns facility management.

## 5. Conclusions

This study introduced a digital twin system and virtual sensor technique to address the limitations of sensor deployment and the high maintenance costs encountered in smart swine barns. In environments where it is difficult to install physical sensors at all locations due to physical constraints and economic burdens, virtual sensors can function as an effective alternative to complement blackout zones and accurately estimate environmental data throughout the entire barn.

From a methodological perspective, this study adopted a hybrid approach that combines spatial interpolation with LSTM-based time-series prediction. Specifically, real sensor data from Zones 1 and 2 were used to define a virtual sensor in the central zone. The initial estimates were obtained through spatial interpolation and then refined using the LSTM model to incorporate temporal patterns. Furthermore, a WebGL-based digital twin visualization environment was developed to provide an integrated platform for intuitively verifying the predicted results of virtual sensor data.

Experimental results demonstrated that the virtual sensor achieved high prediction performance across all variables—temperature, humidity, CO_2_, and NH_3_—with coefficients of determination (R^2^) exceeding 0.95. In particular, the hybrid model produced more stable results for variables with significant spatial heterogeneity, such as CO_2_ and NH_3_, by simultaneously capturing spatial and temporal characteristics. These findings experimentally confirm the practical effectiveness of integrating digital twin and virtual sensor technologies for smart barn environmental management.

By compensating for sensor blackout zones through virtual sensing, this study enabled the acquisition of high-resolution environmental data and the establishment of a reliable predictive model. The digital twin-based visualization environment also allowed operators to intuitively interpret data and assess real-time environmental risks. Additionally, the virtual sensor technology reduces installation and maintenance costs, enabling small- and medium-sized farms to adopt smart barn systems at a lower cost. This advancement can contribute to improving animal welfare, enhancing productivity, and ultimately stabilizing farm income. Moreover, the proposed approach aligns with national policies on digital transformation and low-carbon swine barns management and can support government initiatives to expand smart barn deployment and promote carbon neutrality. As an ICT-integrated swine barns management model, it holds potential to serve as a foundational technology for sustainable agricultural and swine barns innovation at the national level.

The applicability of the proposed framework can be extended to larger or structurally diverse swine barns, as the hybrid model does not rely on a fixed barn geometry. However, facilities with multiple ventilation inlets, multi-level layouts, or highly heterogeneous environmental zones may require additional sensor layers or zoning adjustments to ensure accurate spatial representation. While the present study demonstrates the feasibility of virtual sensing within a two-zone pig barn, future work should examine scalability in larger commercial settings to verify performance under more complex airflow patterns and environmental distributions.

Future research should aim to further enhance predictive performance by incorporating advanced spatio-temporal fusion models, such as Transformers or graph neural networks, and by validating the proposed framework across swine barns with different structural layouts or environmental characteristics. Extending the methodology to environments that exhibit vertical stratification, including multi-level barns or greenhouse systems, may also provide valuable insights into its broader applicability. In addition, integrating WebGL-based visualization with real-time control systems would support the development of more comprehensive and intelligent swine barns-environment management platforms.

## Figures and Tables

**Table 1:** Overview of the research dataset.

Zone	Sensor	Unit	Date	Measurement Interval
1	Temperature, humidity, CO_2_, NH_3_	°C, %, ppm	1 January–31 August 2025	10 min
2	Temperature, humidity, CO_2_, NH_3_	°C, %, ppm	1 January–31 August 2025	10 min

**Table 2:** Specifications of sensors used in the study.

Variable	Sensor Model	Principle/Output	Range	Accuracy (Typ.)
Temperature/ Humidity	SHT3x-DIS	Digital temp & RH (I^2^C)	−40 ~ +125 °C; 0–100% RH	±0.1–0.2 °C; ±1.5–2% RH
CO_2_	MH-Z14A	NDIR, analog/ PWM/UART	0–5000 ppm (up to 10,000 ppm)	±50 ppm ± 5%
NH_3_	ME3NH3	Electrochemical	0–100 ppm	±3 ppm (typ.)

**Table 3:** Prediction performance evaluation results.

Variable	Model	RMSE	MAE	R^2^
Temperature (T_virtual)	LSTM	0.438	0.363	0.970
Temperature (T_virtual)	Hybrid	0.515	0.434	0.959
Humidity (RH_virtual)	LSTM	1.805	0.845	0.978
Humidity (RH_virtual)	Hybrid	1.887	0.960	0.976
CO_2_ (CO_2__virtual)	LSTM	23.348	16.252	0.980
CO_2_ (CO_2__virtual)	Hybrid	22.733	16.780	0.981
NH_3_ (NH_3__virtual)	LSTM	0.392	0.310	0.952
NH_3_ (NH_3__virtual)	Hybrid	0.340	0.223	0.964

**Table 4:** Performance comparison results by algorithm.

Variable	IDW RMSE	LSTM RMSE	Hybrid RMSE	IDW R^2^	LSTM R^2^	Hybrid R^2^
Temperature	0.000	0.438	0.515	1.000	0.970	0.959
Humidity	0.000	1.805	1.887	1.000	0.978	0.976
CO_2_	0.000	23.348	22.733	1.000	0.980	0.981
NH_3_	0.000	0.392	0.340	1.000	0.952	0.964

## Data Availability

The data presented in this study are available on request from the corresponding author. The data are not publicly available due to confidentiality agreements with the collaborating company and restrictions related to joint research.

## Abbreviations

The following abbreviations are used in this manuscript:

AI	Artificial Intelligence
ICT	Information and Communication Technology
IoT	Internet of Things
DT	Digital Twin
IDW	Inverse Distance Weighting
LSTM	Long Short-Term Memory
CNN	Convolutional Neural Network
RNN	Recurrent Neural Network
GNN	Graph Neural Network
STF	Spatio-Temporal Fusion
MAE	Mean Absolute Error
RMSE	Root Mean Square Error
CO_2_	Carbon Dioxide
NH_3_	Ammonia
RH	Relative Humidity
GUI	Graphical User Interface
WebGL	Web Graphics Library
API	Application Programming Interface
JSON	JavaScript Object Notation
